# Evaluating the gut microbiome, dietary patterns, and cognition: a sub-study protocol from the brain health and the gut microbiome study in cognitive decline (bMicrobiome study)

**DOI:** 10.1017/gmb.2026.10024

**Published:** 2026-04-20

**Authors:** Karol Suchowiecki, Patrick G. Corr, Aidan Schurr, Aryan Asemani, Leigh A. Frame

**Affiliations:** 1 Department of Medicine, University of Connecticut Health Center, USA; 2 Department of Clinical Research and Leadership, School of Medicine and Health Sciences, The George Washington University, USA; 3 School of Engineering and Applied Science, The George Washington University, USA; 4 Milken Institute School of Public Health, The George Washington University, USA

**Keywords:** gut microbiome, dietary patterns, Alzheimer’s disease, behaviour change, dietary assessment

## Abstract

The aim of this study is to describe the development and implementation of a novel Readiness to Change Nutritional Habits (RCNH) survey for use along with dietary assessment and gut microbiome profiling in a proof-of-concept study in individuals with early Alzheimer’s disease dementia (eAD), mild cognitive impairment (MCI), and healthy controls (HC). Overall, this methods paper contributes to emerging research examining how behavioural readiness for change can be integrated with dietary assessment and gut microbiome profiling to better understand the microbiome’s influence on the nervous system. This is a sub-study embedded within a multi-prong proof-of-concept, observational study mapping the gut microbiome in 45 participants (15 HC, 15 MCI, 15 eAD) at baseline, 3 months, and 6 months. The parent study collects gut microbiome profiles, dietary patterns, and cognitive assessments. The sub-study develops and administers the 32-item RCNH survey to characterize participants’ readiness to adopt nutritional change. This manuscript reports the RCNH survey, its development process, the sub-study protocol including data collection procedures, and planned exploratory analyses. This protocol presents a novel intervention to assess the gut microbiome, individual dietary patterns, and readiness to make lifestyle changes related to diet.

## Background

### The gut microbiome and cognitive health

The gut microbiota (the community of microorganisms inhabiting the gastrointestinal tract) and the gut microbiome (the microbiota and their collective genetic and functional potential) play a vital role in human health and pathogenesis of disease, including potential associations with cognitive function and Alzheimer’s disease and related disorders (ADRD) (Guinane and Cotter, 2013; Manderino et al., 2017). Research has established that there is a possible protective factor against cognitive decline in the gut microbiota (Harding and Bishop, 2022; Liang et al., 2022). Emerging human and animal data suggest that individuals with cognitive impairment may exhibit distinct microbiome features compared with cognitively healthy peers and that microbiome variation may correlate with established neuropathologic biomarkers (Quigley, 2017; Ferreiro et al., 2023). A recent cohort study by Ferreiro et al. demonstrated that individuals with preclinical Alzheimer’s disease had distinct gut microbiota composition and that change in composition correlated with β-amyloid (Aβ) and tau pathological biomarkers (Ferreiro et al., 2023). Proposed mechanisms that implicate the gut microbiome in cognitive decline include altered gut barrier integrity, immune activation, and downstream neuroinflammation via gut–brain signalling pathways (Quigley, 2017; Hirschberg et al., 2019; Sinha et al., 2024; Warren et al., 2024). Together, these findings support the rationale for longitudinal characterization of microbiome composition in populations across the cognitive continuum.

### Dietary patterns influence gut microbiome composition

Individual dietary patterns influence gut microbiome composition because microbes rely on nutrients from digested food (Warren et al., 2024). Microbiome bacteria use microbiota-accessible carbohydrates (MACs) such as resistant starch and fibre and produce metabolites – including short-chain fatty acids (SCFAs) and secondary bile acids (SBAs) – that may affect the gut–brain axis (Warren et al., 2024). Diets rich in MACs correlate with greater SCFA production (Ayakdaş and Ağagündüz, 2023). SBA and SCFA production is influenced by dietary fat and fibre (Wolf et al., 2022). Changes in SCFAs/SBAs are linked to gut barrier function and inflammation (Warren et al., 2024). SCFAs can cross the blood–brain barrier (BBB), supporting a potential role in Alzheimer’s disease (Silva et al., 2020).

The Mediterranean diet (MedDiet) can change microbiome composition over time (Ticinesi et al., 2024). Short-term interventions report increased bacterial diversity (Barber et al., 2021; Godny et al., 2022; Rejeski et al., 2022). Diet broadly shapes microbiome composition (Zhang, 2022). MedDiet components (e.g., fibre and polyphenols) may increase SCFAs (Nagpal et al., 2019; Khavandegar et al., 2024). A systematic review with five observational studies reports a significant increase in SCFAs following MedDiet adherence (Khavandegar et al., 2024). It is evident that dietary changes lead to changes in microbiota composition and production of microbiota-derived metabolites, which are associated with numerous disease states including brain-related disorders like Alzheimer’s disease.

### Dietary modification requires behaviour change

Changing dietary patterns requires behavioural change, a challenge in addressing individual health (Sutton, 2022). The Transtheoretical Model of Change (TTMC) is a well-established framework to describe and categorize the dynamics of individual health-related behavioural change (Prochaska and DiClemente, 1992; Prochaska and Velicer, 1997). This model emphasizes five phases: precontemplation, contemplation, preparation, action, and maintenance. TTMC was first used in research related to psychotherapy, addiction, smoking cessation, weight loss, and alcohol and substance abuse (Shaffer, 2013).

The first stage of change is precontemplation in which there is no intention to make a change in a particular behaviour at least within the next 6 months (Shaffer, 2013). People in this stage either do not recognize that they have a behaviour that needs changing, or they may recognize that a change needs to be made but feel discouraged after multiple unsuccessful attempts to change in the past. Often in this stage, people deny the need for behavioural changes and may response defensively to discussions about modifying behaviour (Raihan and Cogburn, 2023). The second stage of change is contemplation in which individuals both identify and acknowledge an undesirable behaviour or habit and intend to change it within the next 6 months. This stage is characterized by awareness that a problematic behaviour exists, and the individual is seriously considering change; however, there is uncertainty as to whether the benefits of changing outweigh the costs (Raihan and Cogburn, 2023). The third stage of change is the preparation stage which is characterized by the intention to make a change soon, often within a month (Prochaska and Velicer, 1997). In this stage, people have determined that the pros outweigh the cons of the change, and they typically obtain vital information to make the change such as self-help resources, counselling, and professional advice. The fourth stage of change is action, when an individual makes a specific and overt observable change to their behaviour within the past 6 months (Prochaska and Velicer, 1997). Typically, a change in this category needs to meet a recognized threshold to reduce disease risk; for smoking cessation, reductions in cigarettes smoked may not be considered “action” when the target behaviour is abstinence (Prochaska and Velicer, 1997). For example, a reduction in cigarette use would fall in the preparation stage if the goal were abstinence for 6 months. Lastly, maintenance is the final stage of change where individuals have implemented the change and maintained it for at least 6 months. Some individuals may be pursuing methods to prevent relapse; however, many have the new change fully integrated into their daily routines, which require little to no ongoing effort to maintain (Raihan and Cogburn, 2023). Some researchers recognize “termination” as the last stage of change; however, it is very hard to achieve, and often, there is always minimal effort required to maintain change, placing most people in the maintenance phase forever. Although the process of change is often not linear, and individuals may move bidirectionally between stages over time, the TTMC provides a valuable framework for behavioural change in both the clinical and research settings.

### Need for a nutrition-specific readiness to change survey

Numerous validated surveys have been developed based on this model, including a 12-question readiness to change survey and a 32-question University of Rhode Island Change Assessment (URICA) (McConnaughy et al., 1983; Heather et al., 1993; Henderson et al., 2004). At the time of study initiation, we did not identify a validated instrument designed to measure readiness to change nutritional habits in the context of microbiome-informed dietary recommendations, particularly among individuals with cognitive impairment. We sought to measure readiness to change nutritional habits as participants’ level of “motivation” likely would interfere with their implementation of suggested nutritional changes based on the individualized microbiome report. Therefore, this methods-focused sub-study describes the development and implementation of a novel TTMC/URICA-adapted instrument – the Readiness to Change Nutritional Habits (RCNH) survey – and its integration with dietary assessment, microbiome profiling, and cognitive testing within the parent bMicrobiome study.

### Study objectives

The primary contribution of this manuscript is the development and implementation of the RCNH survey and the protocol for integrating readiness-to-change measurement into microbiome-informed dietary recommendation studies.

This is a sub-study of a larger study seeking to map participant microbiomes to establish gut composition among a population of healthy controls, participants with mild cognitive impairment, and participants with early Alzheimer’s disease dementia. This study specifically seeks to describe the development, adaptation, and implementation of the RCNH survey. In addition, this study seeks to explore the relationship between the gut microbiome and dietary patterns, ultimately to identify educational protocols to encourage individual behaviour change and improve cognitive health.

#### Parent study objective

1. To compare the gut microbiomes of patients with early Alzheimer’s disease dementia, mild cognitive impairment, and healthy controls using diversity as well as genus, species, and strain level differences in composition and function.
2. To document microbiome changes following lifestyle changes in subjects with early Alzheimer’s disease dementia, mild cognitive impairment, and healthy controls

#### Sub-study objective

Phase 1 – Survey Development: To describe the development, adaptation, and implementation of the RCNH survey.

Phase 2 – Survey Piloting: To assess the feasibility of measuring readiness to change alongside dietary assessment, microbiome profiling, and cognitive assessment in HC, MCI, and eAD participants.

## Methods

### Parent study overview

The bMicrobiome Study (NCT06039267) is a proof-of-concept observational study designed to longitudinally characterize gut microbiome composition in healthy controls (HC), mild cognitive impairment (MCI), and early Alzheimer’s disease dementia (eAD). At 0 months, 3 months, and 6 months, participants complete gut microbiome profiling (EzBiome), dietary assessment (DietID), and cognitive assessment (BOCA). Participants receive microbiome-informed lifestyle recommendations at each time point; adoption of these recommendations is voluntary and observed over time.

### Sub-study design

This manuscript describes a methods-focused sub-study embedded within the bMicrobiome parent study (NCT06039267). The parent study is a proof-of-concept observational study mapping the gut microbiome longitudinally in HC, MCI, and eAD participants. The purpose of the present sub-study is to implement a newly adapted RCNH survey and to describe its use alongside data on gut microbiome composition (EzBiome), the Boston Cognitive Assessment (BOCA), and individual dietary patterns (DietID). Planned analysis of associations among RCNH, Diet ID, EzBiome, and BOCA will be exploratory and hypothesis generating. These methods are described in detail below, and a timeline of data collection is present in Table 1.

**Table 1.** Data collection process and alignment

Activity	Baseline	Month 3	Month 6
Gut microbiome profiling	X	X	X
Dietary assessment	X	X	X
Readiness for change survey	X	X	X
Boston Cognitive Assessment (BOCA)	X	X	X

### Study participants

This sub-study recruits males and females stratified into three distinct groups as evaluated by a geriatrician with expertise in cognitive and gut health: healthy controls (*n* = 15), those with mild cognitive impairment (*n* = 15), and those with early Alzheimer’s disease dementia (*n* = 15). Inclusion criteria include ages 50–90, diagnosis of early Alzheimer’s disease dementia, diagnosis of mild cognitive impairment, and healthy controls. There are no study-specific exclusion criteria, aside from participants not meeting the inclusion criteria. Healthy controls are not screened for subjective cognitive decline. The inclusion and exclusion criteria for this study are broad and exploratory, given that this is an early-stage feasibility study. Details are reported under the ClinicalTrials.gov registration for the parent study (bMicrobiome Study, NCT06039267): https://clinicaltrials.gov/study/NCT06039267.

Participant recruitment does not differ in any way between the parent and sub-study. Participants enrolled in the study have a pre-existing diagnosis of MCI or eAD. Diagnoses are confirmed through comprehensive evaluation at the affiliated Memory Clinic in the parent study or by systematic review of external medical records to ensure that diagnostic criteria had been met. Healthy controls are screened with BOCA, to ensure that they do not in fact have MCI.

### Recruitment

Study participants are recruited from around the Washington, DC Metro Area, particularly from an integrative medicine clinic, memory clinic, and general internal medicine clinic which are all affiliated with a large academic medical centre in DC. Potential study participants self-identify to be contacted by the study team. Each participant provides an oral explanation of the study and written consent to review. Concerns and questions are addressed. The potential participants are made aware that this study is entirely voluntary and that they could end their participation at any point. Participants who decide to enrol give consent remotely, as per the decentralized design of the parent study. As study procedures require the completion of online surveys and remote assessments, access to a digital, internet-capable device (e.g., a smartphone, tablet, or computer) is an inclusion criterion. Of note, for participants with early Alzheimer’s disease dementia, caregivers are to be involved in the process of data collection, specifically to understand their role in influencing dietary patterns. These contextual factors are to be considered in data analysis and may be a possible limitation to the dataset.

### Data collection and procedures

Below, we describe the design, implementation, and adaptation of the RCNH survey used for the sub-study. In addition, we describe the BOCA, Diet ID, and Ez Biome instruments, which are used for both the parent and sub-study. BOCA has been validated in MCI and eAD individuals. Diet ID, EZ Biome, and the novel RCNH survey have not been validated in MCI and eAD individuals. Of note, all study participants are to complete all study surveys.

### Readiness to change nutritional habits survey (RCNH)

After a robust literature search for a survey that mapped stages of nutritional change, we found that there was no survey that fit our study objectives. However, there are multiple surveys that map stages of change for quitting smoking, alcohol use, physical activity, and utilization of psychotherapy. We model our novel survey on the validated URICA because of its reliable scoring mechanism that maps survey responses to the appropriate stage of change.

To effectively measure the readiness to change and nutritional habits of our participants, we developed a 32-question survey based on the TTMC and URICA. We chose this model to capture the stage of change at which participants are regarding their desire to improve their nutritional habits. The wording of each statement on the URICA is adapted to discuss nutritional behaviours, and the wording is conserved to resemble the original validated URICA survey as much as possible (Prochaska and DiClemente, 1992; Prochaska and Velicer, 1997). For example, the first question is changed from, “as far as I’m concerned, I don’t have any problems that need changing” to “as far as I’m concerned, I don’t have any nutritional habits that need changing” (McConnaughy et al., 1983; Heather et al., 1993; Henderson et al., 2004). A five-point Likert scale is utilized for each survey statement. We name our survey, “Readiness to Change Nutritional Habits” (RCNH). The full survey and scoring criteria are available as supplemental material. Though our novel survey closely resembles the validated URICA, since it is adapted to fit our question, it is not validated, and we hope to utilize the data we obtain from this protocol to aid in the validation of this survey.

To refine the initial draft of the RCNH, we utilize both expert review and cognitive interviewing based on the six survey design principles described by Corr et al. (2023). Expert review consists of sending the survey to two survey design experts who review the draft survey for (1) adherence to the aims of the study and (2) question clarity. These expert reviewers have over three decades of experience in study design, are both well-published in survey methods, and are internationally recognized as experts in survey research in health and medical fields. Both experts are external to this study and have no other role in the design of instruments, data collection, or analysis. The final stage of the process is cognitive interviewing. This involves recruiting five individuals who resemble the study population. To achieve this purpose, three healthy control participants and two participants with mild cognitive impairment were recruited to discuss the draft instrument. Each participant is given the survey and asked to read each question out loud to the researchers. A think-aloud approach is utilized so that participants can state their thoughts on each survey item, ask questions, and provide recommended language and improvements (Corr et al., 2023). Each of these cognitive interviews generally lasts 30–60 minutes. After this process is completed, the suggested changes made by participants are added to the survey to enhance clarity and language use. This process is intended to make sure the survey wording is clear and understandable; therefore, to aid in this process, we opt for participants resembling the healthy control and mild cognitively impaired group. We omit performing cognitive interviewing with eAD patients as the individuals we identify could not undertake the task of survey editing due to the cognitive load and attention it requires. The final draft of the survey is now part of the bMicrobiome Study. This survey data will be analysed in conjunction with the gut microbiome profiling and dietary assessment data to explore the link between microbiome composition, dietary patterns, and cognitive health.

### Boston Cognitive Assessment (BOCA)

The BOCA is a self-administered computerized cognitive test developed for longitudinal cognitive monitoring. BOCA evaluates multiple domains of cognition including attention, memory, language use, executive function, visuospatial ability, and orientation (Vyshedskiy et al., 2022). In this study, BOCA measures cognitive function across multiple time points (0, 3, and 6 months) and is completed in approximately 15 minutes on a tablet, computer, or smartphone. BOCA’s design minimizes learning effects by utilizing random, never-repeating tasks, making it suitable for repeated measurements over the study duration.

Participants access and complete the BOCA remotely through a secure, HIPAA-compliant platform. BOCA also provides a feasible, reliable cognitive function measure without necessitating in-person visits. Participants receive detailed instructions on accessing and completing the assessment online with study team assistance as needed. Members of the study team will be available to provide technical troubleshooting, step-by-step instructions, and real-time assistance in accessing the tool. Results are collected and stored securely, contributing to longitudinal data for each participant, which is essential for tracking potential cognitive changes over the study period. BOCA is complemented by additional self-report and caregiver assessments to enrich the data on participants’ cognitive and functional status.

### Gut microbiome profiling

Gut microbiome analysis in this study is conducted in an academia-industry-government partnership with EzBiome, the GW High-performance Integrated Virtual Environment (HIVE) Lab, and the National Institute of Standards and Technology (NIST), leveraging 16S rRNA sequencing and functional prediction to assess bacterial microbiome composition and function. For this analysis, a stool sample is collected at home at baseline, 3 months, and 6 months with two consecutive samples integrated into a single EzBiome report at each time point. All visit elements (surveys and stool collection) are completed by participants within 2 weeks from reception of study materials at each time point. The participant receives their EzBiome reports and is advised to consider implementing the suggested lifestyle changes at each time point (baseline, 3 months, and 6 months). Any changes the participant makes (either recommended in the EzBiome report or otherwise) while participating in the study are documented by the study team. At each time point, the stool samples are analysed, offering insights into microbial diversity and composition at the phylum, class, order, family, genus, and species levels as well as prediction of functional capabilities of the gut microbiota.

16S rRNA sequencing enables the identification of key bacterial taxa and patterns associated with cognitive health. For the researchers, the EzBiome report provides detailed data on the microbiome’s diversity index and specific microbial signatures linked to inflammation and gut health. For the participants, the EzBiome report provides tailored feedback based on their microbiome profiles, including personalized lifestyle recommendations intended to promote microbiome balance. These recommendations may include dietary adjustments and lifestyle modifications, with changes tracked to evaluate their impact on gut health and study outcomes over time.

All sample processing and analysis are conducted in a CLIA/CAP-certified laboratory with independent verification by NIST and the HIVE Lab, ensuring high standards for quality and data accuracy. Findings from each time point contribute to the study’s exploration of the microbiome–gut–brain axis, focusing on potential associations between microbiome composition, microbiome function, cognitive health, and participants’ readiness to make dietary changes.

At each time point, participants receive microbiome-informed lifestyle recommendations. Implementation is voluntary. Self-reported dietary and lifestyle changes are documented by the study team, and dietary pattern change is assessed using DietID-derived indices across time points. Details on this study will be published separately and do not have direct bearing on the development of the survey itself (Phase 1 Sub-Study Objective).

### Dietary assessment

DietID is a validated dietary assessment tool used to improve dietary management, streamline data collection, and support clinical optimization. A primary benefit of DietID is its modern, digital platform that has been shown to strongly correlate with common food frequency questionnaires (FFQs), 24-hour recalls, food records, and biomarkers (Katz et al., 2020; Turner-McGrievy et al., 2021; Bernstein et al., 2023; Dansinger et al., 2023; Radtke et al., 2023). This makes the task of collecting dietary data more manageable for researchers and less burdensome for research participants, especially for those with cognitive challenges. DietID software relies on a clear and easy to use interface, which presents the user with a few foundational questions about their age, weight, height, and existing dietary restrictions (e.g., food allergies, religious restrictions). After collecting these baseline data, DietID utilizes a visual, picture-based system, wherein users are asked to select images that most look like their average diet (dietary pattern). This visual approach is designed to evoke a sense of connection between the user and the foods they are seeing. The user is presented with two imagined diets, each made up of numerous images based on the foundational questions, to set a baseline. Then, the participant is asked which set of images is most like their current diet, like an eye exam. Once an image is chosen, DietID recalibrates and produces two new sets of images with slightly varied diets (e.g., swapping butter for oil or whole wheat bread for white bread). This allows many diet permutations to be explored, with the goal of identifying a dietary pattern that is as close to the user’s average intake as possible. Once the final dietary pattern is established, DietID utilizes a large database to generate a report of the approximated daily intake of vitamins, minerals, other nutrients, and key nutritional indices, that is, the Healthy Eating Index (HEI). This report allows for direct comparison with dietary recommendations and provides the name of a dietary pattern to which the intake most closely aligns, for example, the MedDiet. Participants in this study have multiple ways to complete DietID: independently using their own devices (e.g., computers or tablets), with the assistance of a caregiver, or the assistance of members of the research team depending on their level of comfort with technology and cognitive impairment.

DietID is chosen as one of the key data-collecting methods for a myriad of reasons, including its concise format, accurate results, and accessibility for the study population. The web-based, visual nature of DietID avoids the cognitive burdens of long-form surveys and dietary interviews, creating a more approachable assessment that yields more accurate results (Rolstad et al., 2011). The accuracy of the software is important to consider when implementing DietID, since image-based questions can be limiting and not reflective of all possible dietary patterns. Recent and thorough analyses of the software show that DietID’s non-invasive, yet rapid approach is a legitimate substitute for other diet-logging methods, such as the 24-hour dietary recall or FFQ (Radtke et al., 2023). Of note, the dietary data captured by DietID are intended to reflect short-term dietary intake, specifically dietary patterns over a 30-day reference period (Turner-McGrievy et al., 2021).

### Primary outcomes

1. Quantitative measurement of study participants readiness to change from the RCNH survey at 0 months, 3 months, and 6 months
2. Feasibility of administering the RCNH survey alongside DietID in HC, MCI, and eAD participants across three time points (completion rates; need for caregiver/staff assistance)
3. Distribution of RCNH readiness stage/scores across groups and over time

### Secondary outcomes

1. Associations between RCNH scores/stage and DietID-derived dietary indices
2. Associations between RCNH and gut microbiome diversity/composition metrics derived from EzBiome
3. Associations between dietary indices, microbiome metrics, and cognitive measures (BOCA) over time

### Statistical analyses

Analyses prioritize feasibility and descriptive summaries of RCNH administration; inferential analyses are exploratory and hypothesis-generating given the feasibility sample size.

We employed descriptive statistics to summarize demographic and clinical characteristics of participants across the three study groups. Correlational analyses (Pearson or Spearman’s Rho, depending on the distribution of participants) are used to examine associations between readiness to change scores, dietary patterns, BOCA results, and microbiome diversity measures. Finally, we anticipate running exploratory regressions to determine if readiness to change may predict dietary patterns and microbiome diversity while adjusting for possible confounders (e.g., age, sex, cognitive status).

### Hypothesis

This study hypothesizes that participants with greater readiness to change will demonstrate healthier eating patterns as reflected in higher Healthy Eating Index (HEI) scores from DietID. Additionally, we expect that greater gut microbiome diversity as measured by EzBiome reports will reflect a lower degree of cognitive decline.

## Discussion

In this sub-study, we specifically aim to investigate how participants’ nutrition readiness to change influences their implementation of dietary behaviour changes and the potential impact on gut microbiome composition and cognitive markers. By combining both objective data from microbiome analysis and subjective measures such as the RCNH survey, this approach allows for a deeper understanding of how individual readiness may shape dietary habits, ultimately affecting microbiome diversity and function, which are essential for cognitive health.

The findings of this sub-study could have significant implications for both scientific understanding and clinical practice in cognitive health. By exploring the relationship between gut microbiome composition and cognitive decline, and considering participants’ readiness to change dietary behaviours, this sub-study highlights the importance of intention to make behaviour change as a factor that may affect health outcomes. If a strong association between nutrition readiness to change and microbiome composition is observed, it may suggest that personalized dietary interventions informed by both microbiome data and behavioural readiness could enhance adherence and efficacy in managing cognitive health.

Regarding clinical practice and public health interventions, an established link between microbiome composition and cognitive decline could lead to development of clinical guidelines that assess and manage cognitive health through gut microbiome profiling and gut microbiome-directed interventions. Gut microbiome profiling could become part of routine evaluations for patients at risk of cognitive decline, allowing for early intervention and the possibility of prevention. Such assessments could be accompanied by personalized dietary recommendations and lifestyle modifications designed to promote a healthy microbiome, potentially slowing the progression of cognitive decline. For example, such lifestyle modifications may include recommendations to increase fibre intake through fruits, vegetables, and whole grains; to incorporate more plant-based foods; and to reduce the consumption of highly processed foods. These approaches have all been recommended for supporting a diverse gut microbiome and may present useful recommendations for individuals based on their individual gut microbiome (Nagpal et al., 2019; Ayakdaş and Ağagündüz, 2023; Khavandegar et al., 2024).

Furthermore, assessing readiness to change offers valuable insights for designing patient-centred interventions and may be useful in clinical practice to help generate a personalized nutritional and lifestyle change plan. Assessing intentions to make behavioural change is a difficult task; however, the TTMC provides the ability to effectively classify patients into a particular stage of change. With this classification, personalized dietary and lifestyle recommendations can be made that differ based on individual stage of change. For instance, individuals in precontemplation or contemplation stages may require educational support and motivational interviewing, whereas those in the action or maintenance stages might benefit more from specific dietary recommendations aligned with their microbiome profile. This tailored approach could improve adherence to lifestyle changes, potentially enhancing gut health and delaying cognitive decline in at-risk populations.

This study’s findings could pave the way for a personalized approach to cognitive health based on an individual’s unique microbiome profile, baseline cognitive health, readiness to make change, and current dietary pattern. The combination of these methodologies could yield a nuanced picture of how behavioural readiness impacts dietary implementation, which in turn may influence microbiome composition/function and related health outcomes.

### Strength and limitations

This study design includes several strengths that help adequately answer the research question. First, the use of both objective and subjective data enhances the robustness of the findings by limiting potential self-reporting bias, which is a common limitation of survey-based research. Second, the partnership with EzBiome, the HIVE Lab, and NIST allows the use of next-generation metagenomics to measure microbiome speciation and diversity and to infer function with independent verification. Third, the use of DietID for dietary pattern assessment provides an accurate dietary assessment that strongly correlates with traditional methods such as FFQs and even novel approaches such as measuring biomarkers, giving us more confidence in the objectivity of the dietary pattern data. Lastly, RCNH captures changes to participants’ readiness to eat healthier throughout the study period. This survey may be able to answer whether in-depth knowledge of one’s own microbiome leads to any change in desire to make nutritional changes.

Some possible drawbacks to our approach include the use of test kits requiring self-collection of stool samples. This may pose a challenge to many study participants – though, there is no feasible better alternative to this method. Regarding the EzBiome microbiome report, it is important to note that this is for research purposes only at present, and what constitutes a “healthy” microbiome is not well-defined in the current literature. It is also important to note that the gut microbiome is unique like a fingerprint, meaning there are many, many “healthy” gut microbiomes. The behavioural suggestions in the report are not necessarily proven to change or improve gut microbiome health; though, most are considered general good advice, for example, eating more plants (McDonald et al., 2018). Because participation required the use of a digital device (for DietID and RCNH), our sample may be biased towards individuals with greater technological literacy and higher socioeconomic status. This limitation may affect the generalizability of findings. To mitigate these issues, we have study staff ready to assist participants in accessing and utilizing these tools. Further, while we attempt to conserve the language of the URICA, we make changes to the URICA survey applying it to a specific aspect of behaviour change (nutrition) instead of behaviour change broadly. Therefore, our survey is not a validated survey. Validation would strengthen future survey research and is a logical next step. In addition, the current iteration of this survey aims to capture readiness to change at a macro level and does not discriminate what types of food the participant may be adopting. Therefore, on its own, this survey may be very broad and not able to detect granular change; however, this issue is mitigated in this study as the participants presumed nutrition goal is outlined for them in the personalized microbiome report. For example, if a participant’s report shows they need to eat more beans, we can infer they fill out the survey with regard to making that specific lifestyle change. Finally, we would be remiss if we did not acknowledge the critical role of informal caregivers in the support of individuals with cognitive impairment. In the context of this study, however, a person’s dietary habits may be shaped by household roles and gender norms in terms of food purchasing, preparation, and patient care (Russell, 2007; Atta-Konadu et al., 2011).

After completion of this study, we expect the results to demonstrate a correlation between the gut microbiome (composition/predicted function), dietary patterns, and readiness to change nutritional habits. We expect participants with a diet rich in fibre, polyunsaturated fats, and polyphenols, all of which are found in the MedDiet, to have greater microbiome diversity than participants with diets rich in red-meat and simple carbohydrates (Merra et al., 2020). We also expect to see participants who are further along in the stages of change model, as measured by our RCNH, to have healthier dietary patterns and, thus, less dysbiotic microbiomes. However, it is likely that cognitive deficit will play a role in RCNH and DietID results. It may also play a role with their understanding of the EzBiome report and its behavioural suggestions, which would be reflected by a lack of positive nutritional change throughout the study intervals. This may be mitigated somewhat by the inclusion of the caregiver.

### Next steps

Building on the findings of this sub-study, future research should focus on validating the relationships between nutrition readiness to change, dietary behaviours, and microbiome composition in larger and more diverse populations. One priority is to refine and validate the RCNH survey specifically for dietary behaviour change in populations with cognitive impairment. This could enable broader clinical application and allow for a more standardized approach to assessing readiness for dietary change in such populations.

Additionally, future studies should explore the potential long-term impacts of personalized dietary interventions based on microbiome composition and readiness to change. Implementing follow-up periods extending beyond 6 months could provide insights into the sustainability of dietary behaviour changes and their ongoing influence on the microbiome–gut–brain axis. Moreover, examining how individual differences in cognitive status might affect adherence to dietary recommendations will help tailor interventions to meet the unique needs of patients across different stages of cognitive impairment.

Though this study does not assess Apolipoprotein E4 (APOE4) status, addition of APOE4 and other relevant biomarkers to a future iteration of this protocol could provide further insight into the relationship between the microbiome and cognitive decline. Studies show that APOE4 carriers have distinct microbiome phenotypes notably with depletion of protective bacteria (Faecalibacterium, Ruminococcus, and Butyricoccus) and increase in pro-inflammatory bacteria (Alistipes and Bacteroides), which are associated with increase amyloid deposition (Tran et al., 2019; Hou et al., 2021; Wadop et al., 2025). Assessment of microbiome change over time compared between APOE4 carriers and non-carriers would be a worthwhile future step to further establish the significance of APOE4 in its relationship between dementia and the gut microbiome.

Integrating multi-omics approaches, such as metabolomics and transcriptomics, would offer deeper insights into the functional implications of microbiome shifts and how these may impact cognitive health. Finally, expanding the study to assess other behavioural factors, such as physical activity and sleep, would provide a more comprehensive picture of lifestyle modifications and their cumulative effect on both microbiome composition and cognitive outcomes. By addressing these areas, future research could strengthen the evidence base for personalized, microbiome-informed dietary strategies in promoting cognitive resilience.

## Conclusion

This sub-study demonstrates the potential for combining microbiome analysis with readiness to change assessments to inform personalized dietary interventions that may benefit cognitive health. By evaluating how participants’ readiness to adopt dietary changes influences gut microbiome composition and associated cognitive health markers, this research underscores the role of readiness to make behaviour change in achieving health outcomes. We hypothesize that the findings will suggest that personalized interventions tailored to an individual’s microbiome profile and readiness for change could enhance adherence and potentially mitigate cognitive decline.

Our approach, which integrates microbiome sequencing, dietary assessment, and behavioural readiness, offers a promising framework for future studies aiming to promote cognitive resilience through lifestyle interventions. Ultimately, this study lays the groundwork for more targeted, patient-centred strategies in cognitive health care, supporting the growing body of evidence on the gut–brain axis and its implications for neurodegenerative conditions.

## Supporting information

10.1017/gmb.2026.10024.sm001 Suchowiecki et al. supplementary material Suchowiecki et al. supplementary material

## Supplementary material

The supplementary material for this article can be found at http://doi.org/10.1017/gmb.2026.10024.
