# Supplementary figures and images for: Evaluating the gut microbiome, dietary patterns, and cognition: a sub-study protocol from the brain health and the gut microbiome study in cognitive decline (bMicrobiome study)
Source: Gut Microbiome (Camb). 2026 Apr 20;7:e8. doi: 10.1017/gmb.2026.10024 (PMC13200020; doi:10.1017/gmb.2026.10024)

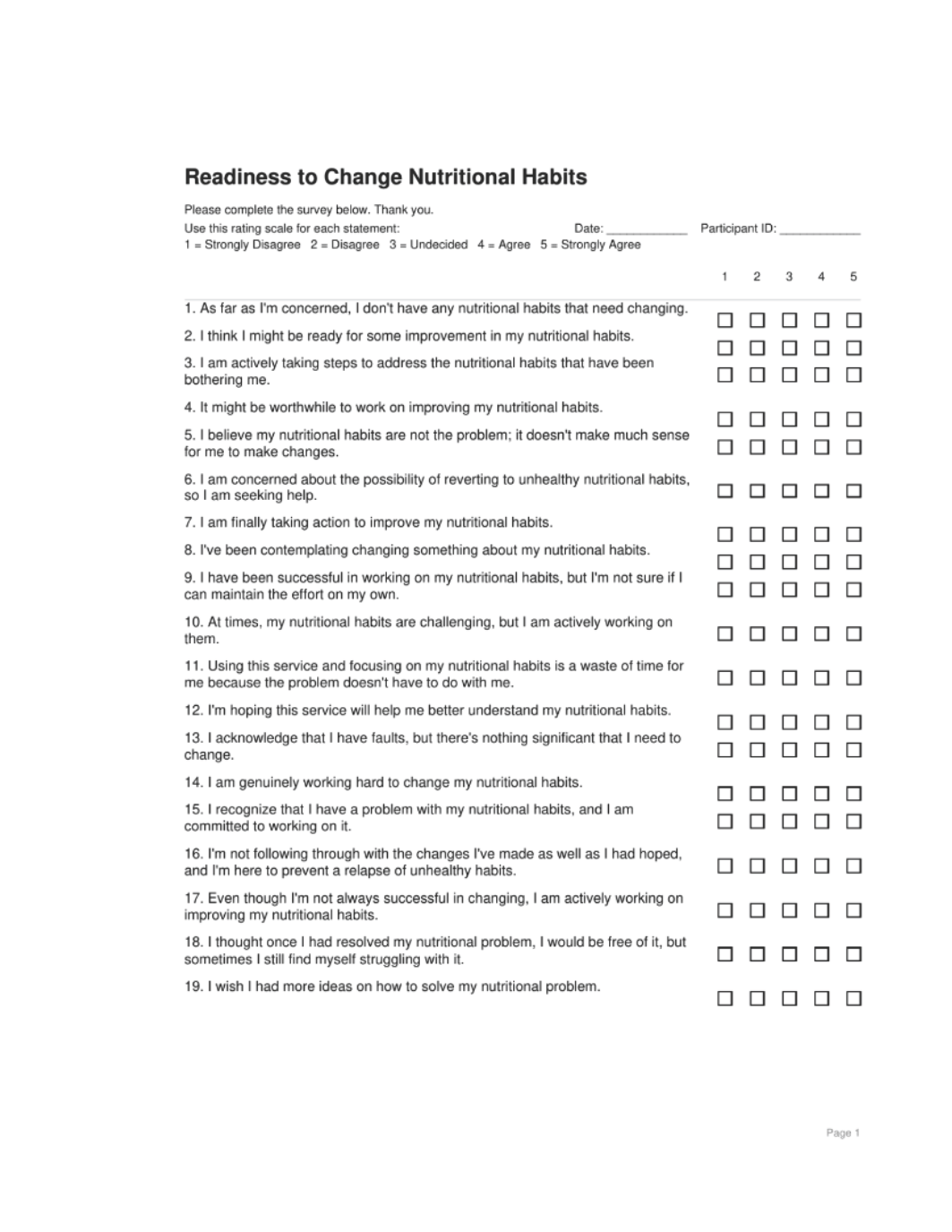

Supplement: Suchowiecki et al. supplementary material [file S2632289726100243sup001.zip › S2632289726100243sup001.tiff]

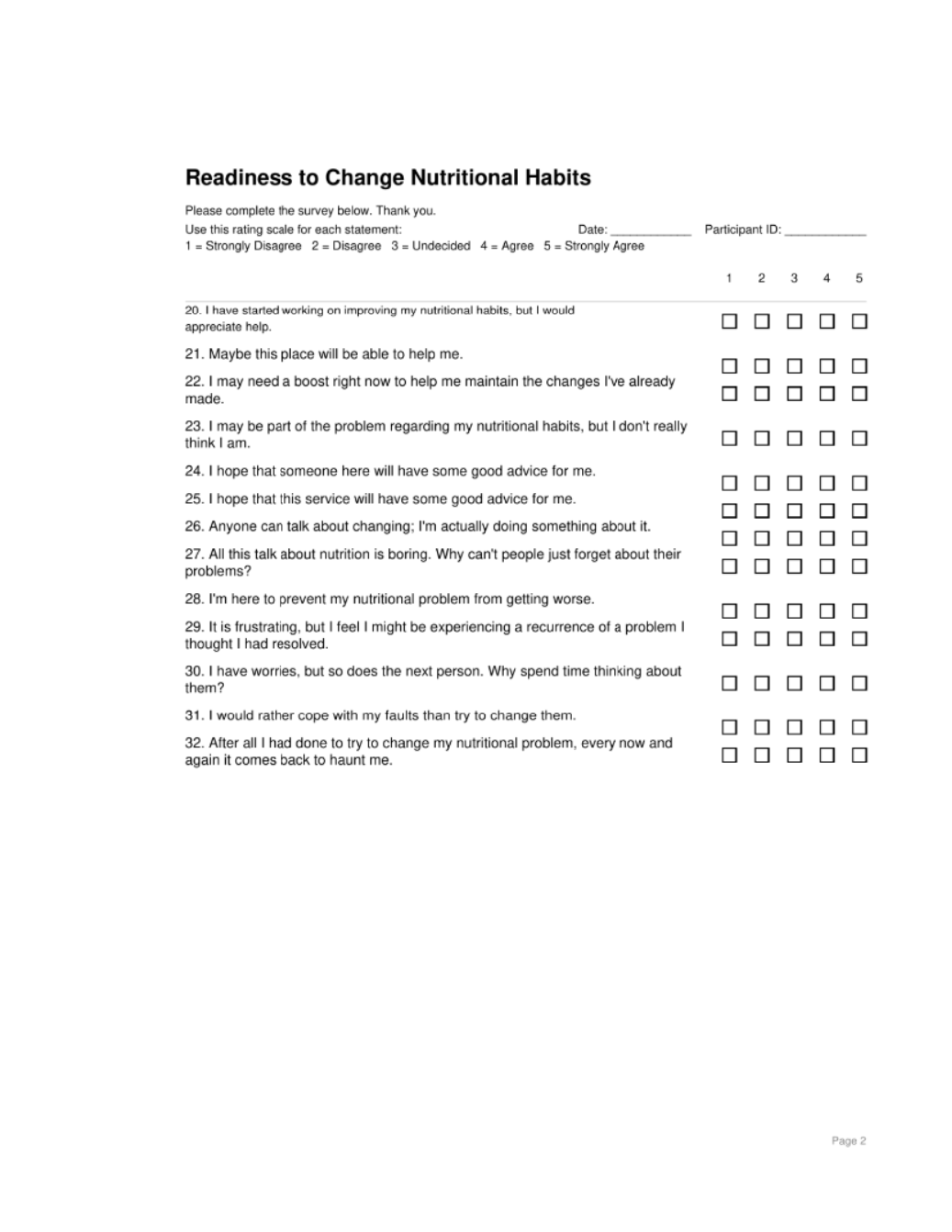

Supplement: Suchowiecki et al. supplementary material [file S2632289726100243sup001.zip › S2632289726100243sup002.tiff]
